# A Sex/Gender Perspective on Interventions to Reduce Sedentary Behaviour in Girls and Boys: Results of the genEffects Systematic Review

**DOI:** 10.3390/ijerph17145231

**Published:** 2020-07-20

**Authors:** Catherina Vondung, Yolanda Demetriou, Anne K. Reimers, Annegret Schlund, Jens Bucksch

**Affiliations:** 1 Department of Natural and Sociological Sciences, Heidelberg University of Education, Keplerstrasse 87, 69120 Heidelberg, Germany; bucksch@ph-heidelberg.de; 2 Department of Sport and Health Sciences, Technical University of Munich, Georg- Brauchle-Ring 62, 80992 Munich, Germany; yolanda.demetriou@tum.de (Y.D.); annegret.schlund@tum.de (A.S.); 3 Department of Sport Science and Sport, Friedrich-Alexander-University of Erlangen-Nuremberg, Gebbertstrasse 123b, 91058 Erlangen, Germany; anne.reimers@fau.de

**Keywords:** sedentary behaviour, screen-time, sitting, girls, boys, children, adolescents, sex, gender, equity

## Abstract

This systematic review aims to evaluate the extent of sex/gender consideration and effectiveness of interventions designed to reduce sedentary behaviour (SB). We searched for randomised or non-randomised controlled trials with the outcome SB and a sex/gender analysis in eleven electronic databases. Sixty-seven studies were included. Sex/gender considerations were qualitatively rated. Sex/gender was reported separately in 44.8% of studies, 14.9% of studies conducted a sex/gender interaction analysis, and 19.4% enrolled either girls or boys. SB was significantly reduced for girls in 16.4%, for boys in 11.9% and for both in 13.4%. No sex/gender intervention effect was found in 38.8%. According to the qualitative rating, studies without significant sex/gender effects reached “detailed” rating twice as often as studies finding a significant intervention effect for either girls or boys, or both. Overall, no clear pattern according to the qualitative rating and in terms of intervention effectiveness can be drawn. The results reveal a lack of sufficient sex/gender information in intervention planning and delivery. Further research should consider analysing sex/gender intervention effects as well as consider sex/gender inclusive intervention planning and delivery.

## 1. Background

While most studies focus on the promotion of physical activity (PA) there is a growing body of evidence that sedentary behaviour (SB) needs to be addressed independently [1]. To date, SB is known for being ubiquitous in high-middle-income countries. Children and adolescents sit up to nine hours per day with a majority of their time spent sedentary (e.g., screen-viewing, sedentary socialising, during school lessons and inactive forms of transport) [2]. High levels of SB can lead to an increased risk of cardio-metabolic disease, all-cause mortality, and a variety of physiological and psychological harms in a later life stage [3,4,5]. SB is a risk factor for mental (e.g., self-esteem, depression, and sleep problems), physical (e.g., obesity, fitness, bone health, and markers of cardio-metabolic risk), and socio-emotional health (e.g., prosocial behaviour, and academic success) in children and adolescents [3,6].

Even small disruptions of SB through PA can have an immediate and long-term positive effect on health in children and adolescents, based on studies of everyday activities of short duration and/or light intensity [7,8]. Any increase in energy consumption through PA and the interruption of SB is particularly relevant for energy-balancing health parameters such as being overweight or metabolising sugar and fat [9,10]. Therefore, the development of interventions aiming to reduce SB is necessary to enhance health.

To date, one of the main correlates associated with SB is sex/gender [11,12]. Based on objectively measured data, girls, on average, show higher levels of SB than their male peers, and this gap widens consistently from childhood into adolescence [2]. For example, in one study, sedentary time increased by 6% in boys and by 10% in girls from age 11 to 12 [12]. Furthermore, girls and boys sit for diverse reasons throughout the day: Boys have been reported to be mostly sedentary for computer-related gaming whereas girls more often sit for social and academic reasons [13]. These differences likely reflect aspects of gender, which operate simultaneously at intrapersonal, interpersonal, institutional and society-wide levels [14]. Gender refers to “the socially constructed roles, behaviours, expressions and identities of girls, women, boys, men and gender diverse people” [15]. Sex/gender theory acknowledges the entanglement of gendered socialisation with sex-linked biological factors [16], and in recognition of this complexity, we use the term sex/gender in this article [17].

Knowing that SB patterns are likely to be maintained in subsequent years and throughout life [18] it is important to understand what kind of SB-reducing interventions work, when, and for whom. Prior systematic reviews and meta-analyses showed that interventions aimed at reducing SB in children and adolescents have small but significant positive effects [19,20]. However, a number of primary studies suggest that intervention effects may differ for girls and boys [21]. Biddle, et al. [19] found that six reviews reported data on sex/gender, and two of these reported differential effects. However, the results were inconsistent across reviews so that conclusions could not be drawn. 

Thus, there is a lack of evidence about the effectiveness of interventions in girls and boys resulting from the insufficient consideration of sex/gender throughout the entire process of intervention planning, delivery, and evaluation [19]. Moreover, there is a lack of a systematic approach to appraise sex/gender within primary studies to appropriately review the sex/gender gap [22]. Although primary research illustrates a need for sex/gender sensitive research [13,23,24], to date there has been no systematic review on SB with special emphasis on the extent to which intervention studies considered sex/gender. Consequently, it is relevant to qualitatively analyse sex/gender in order to avoid inadvertent intervention-generated inequities [23].

Therefore, this systematic review aims to evaluate the effects of interventions aiming to decrease SB in girls and boys, and to appraise the extent to which intervention studies took sex/gender into account.

## 2. Methods 

This systematic review is reported according to Preferred Reporting Items for Systematic Reviews and Meta-Analyses (PRISMA) (Table S1) [25]. The protocol for the review was published previously [22] and also registered with PROSPERO (CRD42018109528). There were no protocol amendments except the GRADE framework was not used due to narrative synthesis of data. The manuscript is part of a systematic review, termed the genEffects project, analysing sex/gender effects of interventions to promote PA and/or reduce SB in children and adolescents [22]. In this paper, we report on studies that focus on the primary outcome SB in order to reach comparability between studies.

## 3. Search Strategy and Eligibility Criteria

A comprehensive literature search was conducted using eleven electronic databases (Cochrane Central Register of Trials (CENTRAL); Ovid Embase; Epistemonikos; EBSCO Eric; Ovid MEDLINE; ProQuest Dissertations & These Global; EBSCO PsycINFO; EBSCO SPORTDiscus; Clarivate Web of Science; clinicalTrials.gov; WHO International Clinical Trials Registry Platform (ICTRP)). The search strategy was based on Cochrane standards and is included for Ovid MEDLINE as Table S2.

The search aimed to identify randomised and non-randomised controlled trials from January 2000 until August 2018 intervening to reduce SB and/ or promote PA in children and adolescents within the average age range of 3 to 19 years. Studies had to be published as peer-reviewed journal articles in the English language, and include a quantified measure of PA and/or SB (objective or subjective measurement). Additionally, all intervention studies must have reported sex/gender disaggregated SB at baseline and/or follow-up, and/or explained how they dealt with sex/gender during the outcome analysis (e.g., sex/gender adjusted analysis), and/or reported that there were no differences in the outcome when looking at sex/gender. The comparators were either an active control group without components promoting PA and/or reducing SB or a control group with no intervention.

## 4. Study Selection and Data Extraction

Records were screened against eligibility criteria by two independent reviewers using Covidence software. All discrepancies were resolved by a third researcher. For each included intervention study, specific details (general study characteristics, sample size stratified by sex/gender and dropout rate, intervention content, approaches and settings as well as outcomes and measurement instruments) were extracted using a piloted data extraction form. Additional information can be found in the systematic review protocol [22].

## 5. Risk of Bias Assessment

Internal validity assessment was carried out independently by two reviewers using the Cochrane risk of bias tool for randomised trials, version 1 [26,27]. Discrepancies were resolved through discussion or adjudication by a third reviewer if consensus was not reached. Primary studies were assessed across the seven domains of the tool that address selection, performance, attrition, detection, reporting, and "other" bias. Each domain was assessed as low, high or unclear risk of bias, with the last category indicating either lack of information or uncertainty about the potential risk of bias. Non-randomised controlled trials were considered to be at high risk of bias for domains related to randomisation. For other risks we included the assessment of baseline differences between intervention and control arm as well as seasonal differences in measurement points and monetary motivational incentives.

## 6. Sex/Gender Assessment

To assess the degree to which sex/gender was considered in intervention studies we used a newly developed sex/gender checklist [22]. The sex/gender checklist consists of 10 items analysing background and concepts, study design, intervention planning and delivery, presentation and interpretation of findings (Table 1). The items are rated using three categories (“basic”, “detailed”, and “no information provided”) defining the extent to which the primary study took sex/gender into account regarding the respective item. A fourth category “not relevant” was used for items that were considered less applicable to single sex/gender studies (items 4, 5, 8 and 9). Some single sex/gender studies have nevertheless provided additional information, which we then rated as “basic” or “detailed”. For the first item, “poor” was also a rating category for those studies that used both terminologies sex and gender interchangeably.

## 7. Data Analysis and Qualitative Synthesis

We undertook a narrative synthesis to analyse differences and similarities between girls and boys in response to interventions, based on statistical method of sex/gender assessment, and the qualitative ratings obtained from the sex/gender checklist. For semi-quantitative analyses, results were stratified into five groups in order to reach comparability between intervention studies. The first two groups are: (1) studies that report intervention effects for girls and boys separately (sex/gender-disaggregated results), and (2) studies that analysed sex/gender intervention effects within interaction analyses (group allocation x time x sex/gender). A third category (3) represents studies that tested for sex/gender differences or similarities in SB at baseline or follow-up measurement or tested for intervention effectiveness via interaction analysis but did not find significant sex/gender differences and did not report the statistical data. Effect sizes for non-significant sex/gender results in so called “tested” studies were not reported. Other groups are (4) studies that adjusted for sex/gender, and (5) studies that enrolled and analysed only girls or boys.

We considered meta-analysis as planned [22] but this was not possible because of the extent of methodological diversity in outcomes and measurement instruments, and a high heterogeneity in effect sizes. The results are presented in two parts. First, we give an overview of all SB study results in terms of overall sex/gender considerations. Then, intervention effects for girls and boys are summarised and for each of the five groups explained above by statistical significance levels in connection with sex/gender rating.

## 8. Results

**Figure 1:**
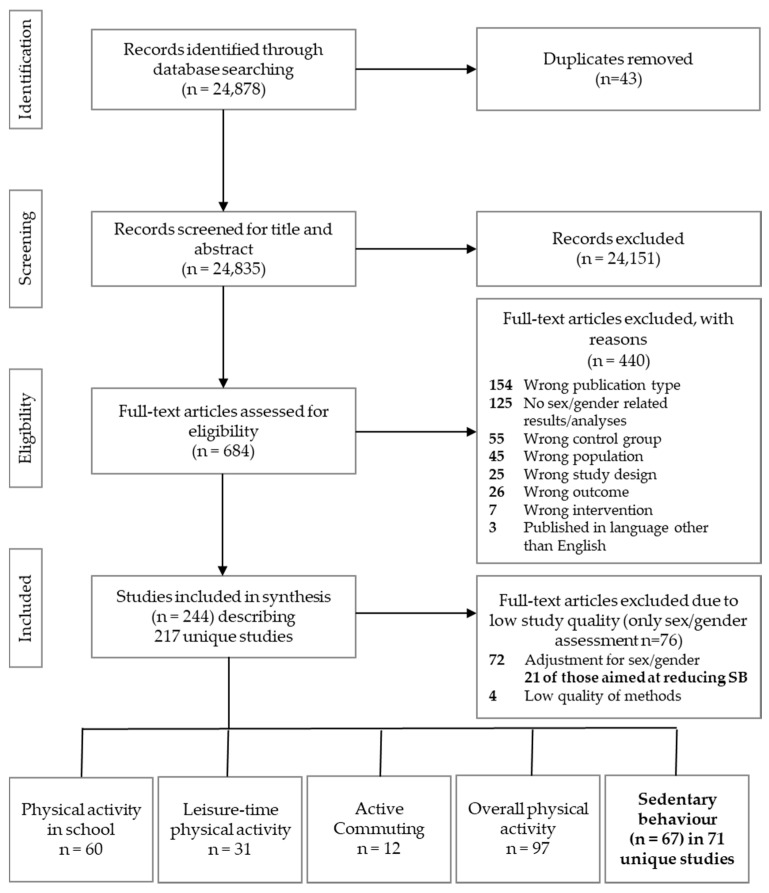
PRISMA Flow Chart.

The literature search generated 24,878 titles of potentially relevant articles of which 67 unique studies (in 71 publications) were eligible for this systematic review (see Figure 1). We identified two publications each for four of the included interventions [28,29,30,31,32,33,34,35].

The majority of primary studies were randomised via clusters (*n* = 38, 56.7%), 17 (25.4%) studies individually randomised participants, and 12 (17.9%) did not use randomisation to assign participants to the intervention or control grouprandomis. School was the most common setting for the interventions (*n* = 54, 80.6%). Community (*n* = 4, 5.9%), home and family (*n* = 3, 4.5%), childcare (*n* = 2, 2.9%), and healthcare (*n* = 2, 2.9%) settings did not occur very often. Two studies were not setting oriented. The target population of 48 (71.6%) studies were preschool children (3 to 6 years) and children (6 to 12 years). Nineteen (28.4%) studies targeted adolescents (12 to 19 years). No study reported on inclusion of gender diverse participants.

### 8.1. Risk of Bias of Primary Studies

**Figure 2:**
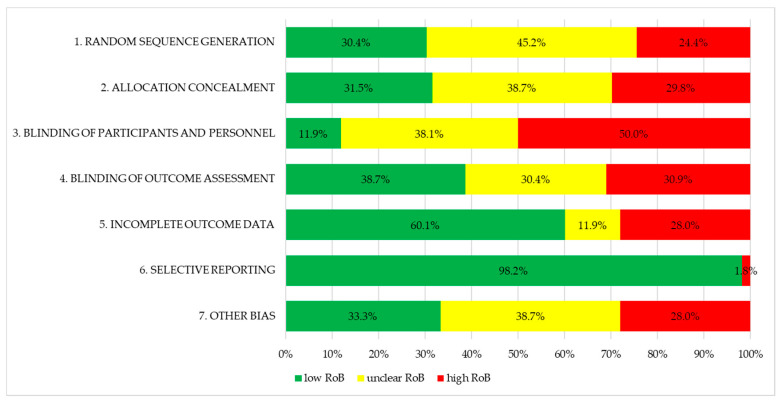
Risk of Bias of all 67 primary studies according to the Cochrane Risk of Bias tool.

Risk of bias of the 67 studies analysing SB as a primary outcome was rated according to the Cochrane Risk of Bias tool (see Figure 2) [26,27]. For random sequence generation, 24% of studies were rated at high risk of bias, and 30% were rated at high risk of bias for allocation concealment. Half of studies were at high risk of bias due to lack of blinding of participants and personnel, 31% lacked blinding of outcome assessors, and a similar proportion of studies (28%) were rated at high risk of bias because of incomplete outcome data. Overall, the majority of studies were rated at high risk of bias in at least one domain

### 8.2. Overall Sex/Gender Analysis of Primary Studies

Comprehensive sex/gender analysis was conducted in 67 intervention studies with SB as their primary outcome. Of these, 30 (50.7%) reported results disaggregated by sex/gender, 10 (14.9%) analysed sex/gender by interaction analyses (group allocation x time x sex/gender), 13 (19.1%) tested for differences or similarities in sex/gender at baseline or follow-up or via interaction analysis but did not find any differences (no effect size shown) and 14 (20.6%) included and analysed only girls or boys in their study. Furthermore, 21 primary studies with the primary outcome SB only adjusted for sex/gender in intervention assessment. These studies have been excluded in further analyses because no assumption about the intervention response in terms of sex/gender can be made.

**Figure 3:**
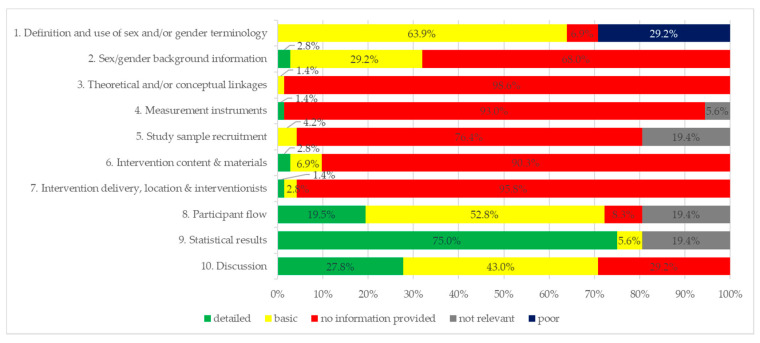
Sex/gender checklist items rated.

Figure 3 provides an overview of proportional sex/gender ratings according to the checklist for each item. Statistical results and discussion (items 9 and 10) were the most common items that were rated as “detailed”. For example, one study analysed intervention effects for girls and boys separately (disaggregated, item 9 rated “detailed”) and stated that “future studies should identify strategies to motivate girls to be physically active” in the discussion (item 10) [36]. The most frequent items that were judged as “no information provided” were in the following categories: background and concepts, study design, and intervention planning and delivery.

In terms of intervention delivery (items 4–7) researchers often do not take sex/gender into account. Therefore, in intervention content, sampling and measuring the outcomes sex/gender are often not considered. In articles where the results show sex/gender specific intervention effects, the researchers are aware of potential differences in girls and boys and furthermore discuss potential underlying reasons (Table 2).

### 8.3. Intervention Effectiveness in Terms of Sex/Gender

In the following, results were stratified according to sex/gender assessment of primary studies.

#### 8.3.1. Sex/Gender Disaggregated SB Studies

Results of 30 sex/gender disaggregated studies reported an effect on SB for girls and boys separately. Of these, 13 (43.3%) studies found non-significant intervention effects for either girls or boys, 9 (30%) found intervention effects that favoured both girls and boys, 3 (10%) found a significant intervention effect for boys only and 5 (13.3%) for girls only. Analysing sex/gender assessment according to the checklist, intervention content and materials were described in terms of sex/gender inclusiveness in two studies (“basic”) and both studies concluded a positive intervention effect for girls and boys [42,58]. One of these studies, Beets, et al. [42], adapted intervention activities specifically for girls (special soccer exercises for girls, dance, etc.) while in Fairclough, et al. [58] intervention lessons were specifically designed for mixed sex/gender groups. In 7 studies, findings were discussed with regard to sex/gender and future directions for interventions were given but no significant intervention effects for neither boys nor girls were found. No other sex/gender checklist item seems to be correlating with intervention effects in the category of disaggregated sex/gender analysis and therefore, no pattern in sex/gender rating was found (Table S3).

#### 8.3.2. Interaction Analysis for Sex/Gender in SB Studies

Possible sex/gender intervention effects were analysed via interaction analysis in 10 primary studies. The identified interaction analysis had to quantify an interaction between girls and boys and group allocation to either intervention or control condition and time before versus after the intervention. In 9 studies, no significant intervention effects for girls or boys were reported. One study reducing childrens’ screen time showed a significantly greater reduction in television time among boys than among girls. This study does not show different sex/gender ratings than the other 9 interaction studies [82]. Additionally, in one study, investigators were aware of the "differential use of materials by girls and boys" and was therefore rated as “basic” in intervention content and materials [55]. The only items rated as “detailed” were participant flow (item 8, 2/10 studies) and statistical results (item 9, 10/10 studies). In 5/10 (50%) interaction studies, results of the intervention were not discussed with regard to sex/gender (Table S4).

#### 8.3.3. Sex/Gender Tested SB Studies

This category represents 13 primary studies that analysed sex/gender differences or similarities in SB at one measurement point (baseline and/or follow-up) but did not find significant intervention effects favouring girls or boys (effect size not reported) and therefore combined the analysis for girls and boys. Due to a lack of data provided, these studies cannot be further summarised in terms of sex/gender intervention effectiveness. Analysing the extent of sex/gender consideration within the checklist, in the introduction to two studies, information on potential differences in SB levels was provided (item 2, [76,95]). For example, it was considered that "boys spend about 2½ h/day and girls somewhat less than 2 h on screen-viewing activities (TV and computer-time combined), and that mean total screen time, as well as TV and computer time, was higher for boys than girls" [95]. The results of the interventions were discussed with regard to sex/gender in 8/13 (72.7%) primary studies (Table S5).

#### 8.3.4. Single Sex/Gender SB Studies

Of 14 single-sex/gender studies, 11 enrolled girls only, and 3 enrolled boys. Of the 11 studies on girls, 7 reported a statistically significant reduction in SB whereas 4 did not. All 3 studies on boys significantly reduced SB. Seven single sex/gender studies that found a significant intervention effect discussed their finding with regard to sex/gender “detailed” (e.g., “Interventions targeting adolescent girls may require additional environmental changes to support health behaviour change” [53]). In summary, single sex/gender studies which show significant intervention effects are rated “basic” and “detailed” on average 2.7 times per primary study. Compared to that, studies without significant intervention effects are rated “basic” and “detailed” on average twice per primary study (Table S6).

#### 8.3.5. Intervention Significance in Relation to the Sex/Gender Checklist

Analysing studies with significant intervention effects across all categories of methods used to address sex/gender, we could not discern a pattern for sex/gender consideration according to the checklist. Twenty-six of the studies that analysed results disaggregated by sex/gender, via interaction, or enrolled and analysed a single sex/gender group, found a non-significant intervention effect in terms of sex/gender. These studies received a “detailed” or “basic” rating on items of the sex/gender checklist twice as often compared to studies that found a significant reduction in SB (*n* = 28) in girls or boys or both sex/gender groups. An additional 13 studies from the category tested found non-significant effects in SB reduction after the intervention. When adding these to all other studies reporting non-significant effects, the increased frequency of “detailed” ratings in the sex/gender checklist disappears.

A second observation in relation to the checklist was identified in studies with interventions that significantly reduced SB in both girls and boys. These reached on average 40.7% more “detailed” ratings across all items of the sex/gender checklist compared to studies with interventions favouring either girls or boys.

## 9. Discussion

This systematic review aimed to determine semi-quantitatively whether the response of the interventions was different or similar for girls and boys, and to qualitatively appraise the extent to which the studies systematically took sex/gender into account in intervention planning, delivery and evaluation. Four interventions significantly reduced SB in girls, 5 interventions in boys. Additionally, 7 single sex/gender studies reduced SB in girls and 3 in boys. Thirty-nine studies did not find differences in intervention effects between girls and boys and 9 studies found an intervention effect for both, girls and boys. No clear pattern in qualitative synthesis of sex/gender consideration in primary studies was found.

Insights of the sex/gender checklist indicated that 76% of studies did not provide information about sex/gender in 4/5 categories. Therefore, it is not possible to give a best practice example out of this systematic review. For sex/gender inclusive interventions it is important to tackle this gap in order to provide valuable information of what works when and why [23]. The aim of the sex/gender checklist is to guide the equal addressing of girls and boys and the consideration of sex/gender throughout the whole research process of intervention studies which encompasses intervention planning, development, implementation, delivery, and evaluation [22]. To the best of our knowledge, our systematic review was the first to exclusively address sex/gender perspectives to explore this research gap in a qualitative manner. In order to reach sex/gender inclusiveness in planning and delivering interventions, our checklist can be a useful and additional tool to reach equity in future health promotion research. This is possible if the checklist is used as a recommendation based on the items displaying how to deal with sex/gender during different aspects within the whole intervention process [23]. 

Moreover, in other systematic reviews assessing SB reductions in intervention studies sex/gender has not been thoroughly considered. However, in terms of the effectiveness these reviews found that it is possible to reduce SB in children and adolescents [18,19], which is also supported by our data. However, it was not an aim of our study to examine the general effect of SB reduction in children and adolescents as we only included studies taking sex/gender into account. Looking at a review of reviews analysing effects of interventions for reducing SB in children and adolescents in terms of sex/gender, the conclusions were inconsistent with only 2/6 reviews reporting a differential effect on girls or boys [19]. Of the included reviews in that study, two reported sex/gender demographic information [99,100] while another concluded that interventional research needs to address this research gap since differences in sex/gender were found and cannot be explained [101]. Another systematic review combined results for girls and boys in the analysis of intervention effectiveness and therefore did not provide further sex/gender information [9]. Therefore, our review adds an important or unravelled perspective.

Our findings revealed that effectiveness of interventions was related to the ratings of the sex/gender checklist in two cases: In 26 intervention studies that found a non-significant intervention effect, sex/gender consideration was twice as high as in significant intervention effects. This could be a result of an in-depth reporting of sex/gender aspects if no effects for the intervention were found as authors might want to explain the absence of significant results. However, this effect gets diluted by 13 studies from the category tested. Reasons for that remain unclear and have to be further analysed in future research. It seems to be positive that 26 included primary studies did not find sex/gender differential effects and considered sex/gender to a greater extent than studies which showed an intervention effect for either girls or boys or both.

Furthermore, a higher extent of sex/gender consideration was also found for studies that had significantly positive effects on both girls and boys compared to studies that favoured either girls or boys. However, 10/19 (52.6%) studies that reported the intervention reduced SB were single sex/gender studies. This has to be kept in mind when interpreting this finding. Reasons for greater sex/gender consideration in individual categories need to be analysed in future research in order to investigate if this might be a consequence of the sex/gender analysis that was carried out or merely a reporting issue. Therefore, we cannot draw an overall conclusion in terms of sex/gender consideration and effectiveness in SB reduction.

Within data analysis we decided to include only studies that allow the assessment of intervention effects in both girls and boys. With this approach it becomes apparent that different options to analyse sex/gender effects have been used and need to be compared to draw an overall conclusion. We categorised studies into five types of dealing with the sex/gender variable. In 21 primary studies with the primary outcome SB, adjustment for sex/gender was done. These studies have been excluded in semi-quantitative analysis as no conclusion in terms of sex/gender effectiveness can be made. Disaggregated study analyses have the potential to analyse intervention effects for girls and boys separately because effect sizes are reported. Single sex/gender studies have a similar potential for analysing intervention effects in terms of sex/gender by analysing either girls or boys, but no conclusion can be made in comparison with the other sex/gender group. Studies that analyse sex/gender within interaction report an effect size for group x time x sex/gender, which seems very valuable as a relation between girls and boys, time of the intervention and group allocation is given. This allows interpretation of girls’ and boys’ response to the intervention. The fourth category, here referred to as “tested”, considered significant sex/gender related effects but did not find any. Thus, the effect size for sex/gender was not reported for this category of studies, which represents 19.4% of all SB studies in the current review. Therefore, it seems that in tested studies the relevance of sex/gender consideration and awareness has not been perceived as important compared to studies that applied other ways to deal with sex/gender in the analysis. In conclusion, the way sex/gender is analysed in primary studies seems to have an influence on sex/gender consideration based on the items of the sex/gender checklist and on the explanatory power of the primary study.

In order to discuss the underlying sex/gender perspective in more detail it is meaningful to put our findings in the context of other reviews taking a similar approach with respect to sex/gender independent of the health behaviour considered. Previous research in the direction of sex/gender awareness appears to be promising as they carried out a qualitative conclusion about sex/gender effectiveness and aim to understand these better in future research [102]. Additionally, a publication gap for sex/gender in primary research was identified [103]. However, beyond assessing the level of effectiveness, sex/gender equity has not been considered in systematic reviews analysing PA promotion and SB reduction even though sex/gender is widely known for having an influence on these behaviours [102,104]. This supports the importance of our approach.

All of our primary studies have been analysed using the Cochrane risk of bias tool which is a current standard for systematic reviews [26]. As we discovered a lack of information in primary studies, we cannot differentiate between low reporting quality and poor methodological quality of the primary studies. This problem persists for the applied sex/gender analysis as well as risk of bias assessment.

The main strength of our review is its comprehensive evaluation of sex/gender in intervention studies on the reduction of SB in children and adolescents [22]. Sex/gender was assessed using a newly developed checklist in an iterative development process and included background and concepts, intervention planning and delivery as well as evaluation. This approach is the first for SB to systematically appraise the sex/gender issue beyond effectiveness.

Limitations of the present systematic review approach are the restriction to English language articles and peer reviewed publication types. As our systematic review includes children and adolescents within an average age range of 3 to 19 years, we recommend further research to analyse the extent of which age moderates possible sex/gender effects. Additionally, even though the calculation of the cumulated effects in SB reduction across all included studies was not the primary aim of this review, it is lacking a meta-analysis due to methodological heterogeneity of primary studies.

## 10. Conclusions

This systematic review represents new findings in the field of sex/gender assessment in health promotion and primary prevention research and leads the field into a promising direction. We encourage researchers to validate the findings by using the checklist as a supplementary material in further interventional research. In sum, sex/gender effects in intervention studies are not sufficiently reported, especially in the development, planning and delivery phases of the studies. Further research should consider analysing sex/gender beyond adjustment or testing and consider sex/gender inclusive intervention planning and delivery to minimise the sex/gender gap in SB.

## Figures and Tables

**Table 1:** Sex/gender checklist: categories, items and their definitions.

Category	Item	Definition
Background and concepts	Definition and use of sex and/or gender terminology	Is the use of sex and/or gender terminology defined in the study?
	Sex/gender background information regarding the research question (e.g., prevalence, strength of association)	Is sex/gender background information regarding the research question taken into account?
	Theoretical and/or conceptual linkages with sex/gender	Is sex/gender linked up with the theory/concept of the intervention?
Study design	Measurement instruments	Are the measurement instruments valid and reliable for sex/gender groups?
	Study sample recruitment	Is the necessity of sampling for sex/gender taken into account?
Intervention planning and delivery	Intervention content & materials (e.g., brochures, leaflets, plans of sessions)	Is/are the intervention content/materials inclusive for sex/gender?
	Intervention delivery, location & interventionists	Is the intervention sex/gender-inclusive regardless the modes of intervention delivery, location and the person carrying out the intervention (instruction/training of implementing persons to be aware of sex/gender-inclusive aspects such as sex/gender-inclusive language)?
Presentation of findings	Participant flow	Is a participant flow chart provided that takes sex/gender into account according to the CONSORT Statement (eligibility, estimation of sample size (baseline), dropout rates (post-test, follow-up))?
	Statistical results	Are sex/gender differences and/or similarities described regarding the outcomes?
Interpretation of findings	Discussion	Are the findings reflected with respect to sex/gender?

**Table 2:** Individual rating of all primary studies according to the sex/gender checklist.

First Author	Reference	Year of Publication	Rating ^1^ of Items ^2^ of the Sex/Gender Checklist									
			1	2	3	4	5	6	7	8	9	10
Aceves-Martins, M.	[37]	2017										
Adab, P.	[38]	2018										
Andrade, S.	[39]	2014										
Annesi, J.J.	[40]	2013										
Bakhoya, M.	[41]	2016										
Beets, M.W.	[42]	2015										
Bergh, I.H.	[43]	2014										
Bhave, S.	[44]	2016										
Breslin, G.	[45]	2012										
Carlin, A.	[46]	2018										
Carson, R.L.	[47]	2014										
Cradock, A.L.	[48]	2016										
Cronholm, F.	[49]	2017										
de Barros, M.V.	[50]	2009										
De Coen, V.	[51]	2012										
De Craemer, M.	[52]	2014										
Dewar, D.L.	[53]	2014										
Duncan, S.	[54]	2011										
Engelen, L.	[55]	2013										
Fairclough, S.J.	[56]	2006										
Fairclough, S.J.	[57]	2013										
Fairclough, S.J.	[58]	2016										
Filho, V.C.B.	[59]	2016										
Grydeland, M.	[60]	2013										
Haerens, L.	[61]	2006										
Harrison, M.	[62]	2006										
Jago, R.	[63]	2006										
Jago, R.	[64]	2015										
Kobel, S.	[65]	2014										
Lanckriet, S.	[66]	2017										
Laukkannen, A.	[67]	2015										
Lawlor, D.A.	[28]	2016										
Llauradó, E.	[36]	2018										
Lubans, D.R.	[68]	2012										
Lubans, D.R.	[69]	2016										
Meier, M.D.	[70]	2007										
Morgan, P.J.	[71]	2018										
Mendez-Gimenez, A.	[72]	2017										
Murillo Pardo, B.	[31]	2016										
Ni Mhurchu, C.	[73]	2008										
Nyberg, G.	[74]	2015										
Nyberg, G.	[75]	2016										
O’Dwyer, M.V.	[76]	2012										
Okely, A.D.	[77]	2017										
Parrish, A.-M.	[78]	2016										
Pate, R.R.	[79]	2016										
Patrick, K.	[80]	2006										
Reilly, J.J.	[81]	2006										
Robinson, T.N.	[82]	2006										
Rosenkranz, R.R.	[83]	2010										
Sacchetti, R.	[84]	2013										
Salmon, J.	[85]	2008										
Salmon, J.	[86]	2010										
Sebire, S.J.	[87]	2018										
Simon, C.	[88]	2004										
Smith, J.J.	[89]	2014										
Smpokus, E.A.	[90]	2010										
Taylor, S.L.	[91]	2018										
Telford, R.M.	[92]	2016										
Toftager, M.	[93]	2014										
van Nassau, F.	[33]	2014										
Verbestel, V.	[94]	2015										
Verloigne, M.	[35]	2015										
Vik, F.N.	[95]	2015										
Weaver, R.G.	[96]	2017										
Weaver, R.G.	[97]	2018										
Young, D.R.	[98]	2006										

^1^■  = detailed; ■ = basic; ■ = no information provided; ■ = not relevant; ■ = poor; ^2^ Checklist items 1–10, as numbered in the table, are: 1, Definition and use of sex and/or gender terminology; 2, Sex/gender background information (e.g., prevalence, strength of association); 3, Theoretical and/or conceptual linkages with sex/gender; 4, Measurement instruments; 5, Study sample recruitment; 6, Intervention content & materials (e.g., brochures, leaflets, plans of sessions); 7, Intervention delivery, location & interventionists; 8, Participant flow; 9, Statistical results; 10, Discussion.

## Supplementary Materials

The following are available online at https://www.mdpi.com/1660-4601/17/14/5231/s1, Table S1: PRISMA 2009 Checklist, Table S2: MEDLINE search strategy, Table S3: Summary of sex/gender disaggregated SB studies, Table S4: Summary of SB studies with interaction analysis for sex gender, Table S5: Summary of sex/gender tested SB studies, Table S6: Summary of single sex/gender SB studies.
