# Supplementary material for: A Sex/Gender Perspective on Interventions to Reduce Sedentary Behaviour in Girls and Boys: Results of the genEffects Systematic Review
Source: Int J Environ Res Public Health. 2020 Jul 20;17(14):5231. doi: 10.3390/ijerph17145231 (PMC7400439; doi:10.3390/ijerph17145231)
Supplement: Supplementary file 1 [file ijerph-17-05231-s001.zip › Table S3_disaggregated SB studies.pdf]

Table S3. Summary of sex/gender disaggregated SB studies

Author, Year	Design, Country, Duration of the interventio n	Age (M±SD), Sample Size (n_{IG} (%male) n_{CG}(%male)), Setting	intervention description	task of control group	aim of the study	SB measurem ent (measure used), outcome reported	Interventi on effect	Sex/gender checklist rating of items													
								1	2	3	4	5	6	7	8	9	10				
Aceves-Martins, M., 2017	cluster RCT, Spain, moderate term	Age_{IG}=14.69±0.75<br>Age_{CG}=14.63±0.78,<br>n_{IG}=170 (50,6% male)<br>n_{CG}=223 (47,5% male),<br>School	Intervention<br>- Training of adolescent challenge creators on social marketing principles and healthy lifestyle theory<br>- 90min/week, 24 weeks activity design sessions, conducted by health promotion and communication specialists; e.g. increase of PA<br>- Challenge creators present intervention in 11 participating classrooms: explained study, provided social media information, invited peers to provide suggestions for activities<br>- implemented 10 activities over 12 months	no intervent ion	increase fruit and vegetable consumpti on and PA, reducing SB	subjective (questionn aire), screen time ≤ 2h/week	⊕⊕;<br>$M_{baseline}$♂= 11.6%<br>$M_{follow-up}$♂= 36.9% (p< 0.01)<br><br>$M_{baseline}$♀= 11.9%<br>$M_{follow-up}$♀= 28.6% (p= 0.01)											2	2	6	0
Beets, M. W., 2015	cluster RCT, USA, moderate term	Age_{IG}=7.9±1.8<br>Age_{CG}=7.9±1.9,<br>n_{IG}= 763 (52,4% male)<br>n_{CG}=223 (51,4% male),<br>School	Intervention<br>- Strategies: following schedule of daily programming, allocated time for PA (60min/day), staff PA-related training (workshop for 3 hours)	no informati on provided	moderate to vigorous physical activity	objective (accelerom eter), time spent in sedentary (min/day)	⊕⊕; OR♂=-5.1 (CI -7.9; -2.4)<br>OR♀=-2.5 (CI -5.2; 0.2)											1	3	5	1
Bergh, I. H., 2014	RCT, Norway, long term	Age_{IG}=11.2±0.26<br>Age_{CG}=11.2±0.27,<br>n_{IG}= 908 (50,4% male)<br>n_{CG}=223 (52,2% male),<br>School	Intervention<br>- targeting energy-balance behaviors<br>- development of health weight promoted through targeting changes screen-behaviour	no intervent ion	screen behaviors	subjective (questionn aire), weekday and weekend	0, not significant (p♀=0.09, p♂=0.83)											4	1	5	0

			- individual, group and environmental strategies to promote a healthy development during school hours and leisure time			TV-viewing															
Bhave, S., 2016	CT, India, long term	male Age_{IG}=9.6<br>female Age_{IG}=9.8<br>male Age_{CG}=9.2<br>female Age_{CG}=9.4,<br>n_{IG}= 304 (47.7% male)<br>n_{CG}=374 (57,22% male),<br>School	Intervention<br>- Increasing PA sessions to 6 per week<br>- Training teachers to engage children in daily yoga-based breathing exercise<br>- Offering attractive PA sessions during holidays<br>- Health education programme, with weekly age-appropriate interaction 1-h sessions teach children about importance of healthy life<br>- Workhops for teachers	no intervention	reduce adiposity and increase physical fitness	objective (accelerometer), watching TV	⊕⊕; $p<0.001$											3	1	6	0
Carson, R. L., 2014	CT, USA, moderate term	Age= 9-14 years,<br>n_{IG}=95<br>n_{CG}=34,<br>School	Intervention<br>- 6h experimental workshop where PE teachers received information and skill-based training, develop and action plan to implement a new and achievable PA opportunity<br>- Support for teachers	wait-list-control-group	implementing new PA programs	objective (questionnaire), SB in school	⊕⊕; control group spend more sedentary time during school from baseline to post by ♂10.3 min/day (CI 4.9, 14.6) and ♀12.9 min/day (CI 7.9, 18.0) compared											2	1	7	0

							to intervention group														
Cronholm, F., 2017	CT, Sweden, long term	male Age_{IG}=7.8 ±0.6<br>female Age_{CG}=7.9±0.7<br>male Age_{CG}=7.9±0.6<br>female Age_{CG}=7.9±0.7,<br>n_{IG}= 106 (62,23% male)<br>n_{CG}=88 (56,82% male),<br>School	Intervention<br>- Extra PE classes: increase PE curriculum of 200min per week; daily 40min classes during 5 school days all school weeks<br>- Ordinary teachers supervised PE classes that included a variety of different activities, such as ball sports, running, climbing and jumping	no intervention	PA and SB	subjective (questionnaire), sedentary time	0; not significant											3	2	5	0
de Barros, M. V., 2009	cluster RCT, Brazil, moderate term	Age=18.4±2.3,<br>n_{IG}= 474 (37,1% male)<br>n_{CG}=515 (43,3% male),<br>School	Intervention<br>- Healthy diet and PA education<br>- Assess the number of days students accumulate at least 60min of MVPA in the past 7 days in a typical week<br>- Increasing obesity trend among youth (PA and eating habits) increase number of days / week on which students accumulate 60min MVPA<br>- Reduce proportion of subjects who did not accumulate 60min MVPA	no information provided	PA for highschool students in Brazil	subjective (questionnaire), proportion of PA inactive (0d/week)	⊕⊕;<br>♂$CG_{baseline}$: 8.8<br>CG_{post}: 11.5<br>$IG_{baseline}$: 11.1<br>IG_{post}: 5.8<br>Intervention x Control (postintvention); p=0.05<br>CG (Baseline) x CG postintervention; p=0.36<br>CG postintervention x CS postintervention; p=0.13											1	1	7	1

							♀ $CG_{baseline}$= 319.5 CG_{post}= 21.6 $IG_{baseline}$: 17.8 IG_{post}= 13.5 Intervention x Control (postintvention); p=0.01 CG (Baseline) x CG postintervention; p=0.63 CG postintervention x CS postintervention; p=0.19														
De Craemer, M., 2016	cluster RCT, Belgium, moderate term	Age_{IG}=4.4±0.6 Age_{CG}=4.3±0.6 n_{IG}= 529 (54,6% male) n_{CG}=330 (53,9% male), Child Care/ Kindergarten	Intervention<br>Interventions implemented by kindergarten teachers; teachers had 2 training sessions (researchers explain goals and material)<br><br>ToyBox Teachers' guide (e.g., background information, definition of PA etc.), classroom activity guides, newsletters, tip-cards, posters, kangaroo hand puppet	no intervention	decreasing SB	objective (accelerometer), SB, subjective (questionnaire), TV weekend and week	⊕♀; TV weekend ß=−9.98 (CI -19.74; -0.23; p= 0.04)											4	1	5	0

Fairclough, S. J., 2016	CT, UK, short term	Age_{IG}=10.7 ± 0.6<br>Age_{CG}=10.7±0.6<br>n_{IG}= 73<br>n_{CG}=66,<br>School	Intervention<br>- Teachers received 4 hours of training in the delivery of curriculum resource<br>- Worksheets, weekly lesson plans, homework tasks, lesson resources, CD-ROM<br>- Key component: "Move more and sit less"	no intervention	PA	objective (accelerometer), sedentary time in PE class	⊕⊕; T0 vs. T1 d♀=1.08 ($p<0.001$)<br>d♂=1.30 ($p<0.001$)											1	1	7	1
Grydeland M., 2013	RCT, Norway, long term	Age_{IG}=11.2±0.3<br>Age_{CG}=11.2±0.3,<br>n_{IG}= 215 (46% male)<br>n_{CG}=485 (40% male),<br>School	Intervention<br>- Team of teachers involved to promote healthy developmen<br>- Yearly kick-off meeting with teachers to ensure they knew rationale and were familiar with intervention components<br>- Teachers received external support in form of short monthly e-mail from HEIA-study-group<br>- Parental fact sheets with information about screen time	no intervention	physical activity	objective (accelerometer), sedentary activity	⊕♀; M= -22 (CI -43; -2; p= 0.03)											3	2	5	0
Haerens, L., 2006	RCT, Belgium, long term	Age=13.1±0.8, (61,9% male), School	Intervention<br>- More opportunities to be physically active during school breaks, at noon or after-school hours<br>- Extra PA on after-school hours and at noon<br>- Extra sport materials (box)<br>- Personal PA advice<br>- Parents interactive group meeting on PA and relationship with health	no information provided	physical activity and healthy eating in middle schools	objective (accelerometer), sedentary time (min/day)	⊕♀; $F_{condition}$ post 1-post 2= 2.3<br>$F_{condition}$ pre – post 2= 0.9											3	2	5	0

Lawlor, D.A., 2016	cluster RCT, UK, moderate term	Age_{IG}=9.5±0.3 Age_{CG}=9.5±0.3, n_{IG}=1064 (47.4% male) n_{CG}=1157 (47.4% male), School	Intervention<br>- 16 lessons: 1 with 60min PA per day and 1 with reduced sedentary time<br>- Homework assignments with parental involvement for activities at home	no intervention	improve levels of physical activity and diet in 9- to 10-year-olds	objective (accelerometer) ,screen viewing minutes per day	0, not significant											3	1	6	0
Llauradó, E., 2018	RCT, Spain, moderate term	Age=13-15years, n_{IG}= 349 n_{CG}=154, School	Intervention<br>- Improve nutritional food selection, healthy habits<br>- Encourage PA<br>- Improve toward a healthy lifestyle<br>- Encourage the intake of healthy drinks<br>- Decrease the consumption of candies and pastries while increasing the intake of fresh fruits and nuts	no information provided	to verify the sustainability of t lifestyles in 13- to 15-year-old adolescents	subjective (questionnaire),TV and/or video games h/day ≤ 2 h/day	0, not significant											1	2	7	0
Meier, M.D., 2007	RCT, USA, short term	Age_{IG}=5-12years $Age_{CG=}$ 13-18years, n_{IG}= 190 (47 male) n_{CG}=97 (50,5% male), Home	Intevention<br>- changes in lifestyle	The unlimited viewing group (=CG) was instructed to continue living the same lifestyle without modifying behavior	leisure-based screen time and physical activity in families	objective (pedometer),TV time	0, not significant											3	1	6	0
Murillo Pardo, B., 2016	CT, Spain, long term	Age=12-15 years, n_{IG}= 368 (58,4% male) n_{CG}=314 (49% male), School	Intervention<br>- Curricular channel: action plan which classroom teachers received training gin PA promotion and, in turn, involves students in	no information provided	moderate-to vigorous physical activity and	objective (accelerometer), screen time	0, not significant											2	1	7	0

			the design of PA-related activities and inactivities<br>- Non-curricular channel: regular information dissemination activities through which school members, families, community were kept updated about intervention and participation in institutional programs and special PA events in the community		sedentary time																	
Nyberg, G., 2015	cluster RCT, Sweden, moderate term	Age=6.2,<br>n_{IG}= 129 (52,7% male)<br>n_{CG}=112 49.1% male),<br>School	Intervention<br>- Health information brochures for parents<br>- Motivational interviewing<br>- Classroom activities (target children's knowledge, attitudes and preferences and parent's role modelling)	wait-list-control-group	prevention of overweight and obesity in six-year-old children	objective (accelerometer), sedentary time all week	⊕♂;<br>group x time: 2.7 (CI 0.29; 5.0; p= 0.03)												2	1	6	1
Parrish, A.-M., 2015	cluster RCT, Australia, moderate term	Age_{IG}=14-13 years,<br>n_{IG}= 891 (48,71% male)<br>n_{CG}=691 (51,2% male),<br>School	Intervention<br>- Involving physical, promote break time PA<br>- Maximum 5 min sitting period at recess and 10 min at lunch, minimum 5 extension of each break period, a revision of each schools policies to encourage maximum access to sporting fields during break times	wait-list-control-group	increase break-time physical activity levels among primary/elementary school children	objective (observation), sedentary time	0, not significant												2	2	6	0
Pate, R. R., 2016	cluster RCT, USA, moderate term	Age_{IG}=4.59±0.4<br>$Age_{CG=}$ 4.5±0.4,<br>n_{IG}= 188 (48,9% male)<br>n_{CG}=191 (51,8% male),<br>School	Intervention<br>- Instruction channel: activities that girls and young women typically enjoy were offered in addition to competitive sports and other traditional PE activities	wait-list-control-group	minutes/hour of moderate-to vigorous-intensity physical activity (MVPA)	objective (accelerometer), sedentary time (min/h)	0, not significant												4	1	5	0

			- Environment channel: role modelling by faculty and staff, increased communication about PA, promotion of PA by school nurse, family-based activities																		
Patrick, K., 2006	RCT, USA, moderate term	Age=12.7±1.3, n_{IG}= 424 (47,64% male) n_{CG}=385 (46,49% male), health care	Intervention<br>- Computer-supported intervention; coupled with a printed manual to take home<br>- Parent intervention intended to help parents encourage behavior change attempts<br>- 16-section printed Teen Guide, mail, and telephone support changes in target behaviour	control group received sun protection behavior program	participation in moderate to vigorous PA, limitations on SB, dietary intake	subjective (questionnaire),sedentary time per day	⊕⊕; p=0.001											3	2	5	0
Reilly, J. J., 2006	RCT, Scotland, moderate term	Age_{IG}=4.2±0.3 Age_{CG}= 4.1±0.3, n_{IG}= 268 (47,8% male) n_{CG}=277 (52,3% male), Child Care/ Kindergarten	Intervention<br>- Nursery elements: 30min sessions of PA<br>- Home elements: family resource packs with information material, health education leaflets, posters focusing on increasing PA through walking and playing	wait-list-control-group	reducing body mass index, PA	objective (accelerometer), sedentary time	0, not significant											2	2	6	0
Sacchetti, R., 2013	RCT, Italy, long term	Age=8-11 years, n_{IG}= 212 (51,8% male) n_{CG}=216 (52% male), School	Intervention<br>- Physical activity program in gym, classroom and schoolyard<br>- Daily physical activity<br>- consisted of at least 30 minutes of physical exercise per day, divided between the schoolyard (vigorous<br>- activity) and the classroom (moderate activity)	no intervention	improving physical abilities	subjective (questionnaire), daily sedentary time	⊕⊕; ♂10 % vs. ♀12 % reduction (pre-intervention versus post-intervention p< .05; intervention versus control											1	2	6	1

							group p< .01)														
Salmon, J., 2008	RCT, Australia, moderate term	Age=10.8years, n_{IG}= 66 (49,3% male) n_{CG}=62 (49,2% male), School	Intervention<br>- Intervention 1: Behavioural modification (e.g. Increasing PA, the home environment, community environment, intelligent viewing and decreasing sedentary behaviour)<br>- Intervention 2: Fundamental movement kills (e.g. dodge and kick, vertical jump, run and throw)<br>- Intervention 3: Behavioural modification and fundamental movement skills	no intervention	reduce time spent in screen behaviours, promote participation in PA	subjective (questionnaire), playing electronic games	0, not significant											1	2	6	1
Salmon, J., 2010	RCT, Australia, short term	Age=10.3±0.62, n_{IG}= 467 n_{CG}=223 (42% male), Home	Intervention<br>- Behaviour change strategies: self-monitoring, behavioural contracting and budgeting of television viewing<br>- Lessons: introduction of PA and heath, patterns of TV viewing, selective TV viewing, decision-making skills, identifying alternative strategies, Switch-2-Activity games and pedometer	wait-list control group	screen-based behaviours	subjective(questionnaire),weekend screen time	⊕♂, coefficient = -0.62 (CI -1.15, -0.10; p=0.02; Cohen's d= 0.2), approx. 20-min difference in change scores over time between intervention and control groups.											3	1	6	0
Simon, C., 2004	cluster RCT, France, moderate term	Age_{IG}=11.6±0.6 Age_{CG}= 11.7±0.7, n_{IG}= 475 (46,3% male) n_{CG}=479 (51,8%	Intervention<br>- Promote PA inside and outside school	no intervention	physical activity and sedentary	subjective (questionnaire), TV/computer video	⊕⊕; ♂ 2003 vs. 2002 OR= 0.92 (CI 0.77, 1.10)											4	1	5	0

		male),<br>School	- Knowledge, attitudes, beliefs and motivation towards PA<br>- Social support by parents, peers, teachers and PA instructors<br>- Environmental -physical, structural and institutional- conditions for PA<br>- The primary study comparison will be made between the individual students of the four intervention and the four control schools (The control group follows the usual health curriculum and PE but none of the ICAPS<br>- interventions.) with respect to changes from the beginning of the study to the end of the intervention period. Changes in body mass index (BMI) and percent body fat (PBF) are the primary end points. Secondary end points include changes in different cardiovascular risk factors (waist circumference, blood pressure, serum insulin and lipids, haemostasis factors), PA and sedentary levels, and also knowledge, attitudes and motivation towards PA. In this study, the changes after 6 months of intervention in some behaviours and psychological variables are presented.		behaviour're.	games > 3h/day	IG vs. CG OR= 0.52 (CI 0.35, 0.76) $p<10^{-3}$<br><br>♀ 2003 vs. 2002 OR= 0.65 (CI 0.47, 0.90) IG vs. CG OR= 0.54 (CI 0.38, 0.77) $p<10^{-3}$														

Telford, R. M., 2016	cluster RCT, Australia, long term	male Age$_{IG}$=8.1±0.4<br>female Age$_{IG}$=8.3±0.3<br>female Age$_{CG}$= 8.1±0.4<br>male Age$_{CG}$=8.2±0.3,<br>n_{IG}= 432 (51,9% male)<br>n_{CG}=375 (49,6% male),<br>School	Intervention<br>- PE taught by a Bluearth specialist (university trained and qualified PE teaches with further specialised training in Bluearth approach); wihich amounted to 90min of the mandatory weekly PE	no intervention	increase PA or fitness	objective (pedometer and accelerometer), sedentary time (mins/day)	0, not significant											1	3	5	1
van Nassau, F., 2014	CT, Netherlands, long term	n_{IG}= 1002<br>n_{CG}=484,<br>School	Intervention<br>- Reducing intake of sugar containing beverages<br>- Reducing intake of high-energy snacks/sweets<br>- Reducing screen time<br>- Increasing levels of physical activity<br>- Consuming a daily breakfast	no intervention	to evaluate the intervention effects of DOiT-implementation programme on adolescents' adiposity and EBRBs (energy balance-related behaviours)	subjective (questionnaire), sedentary behaviour screen based	0, not significant											1	3	5	1
Verbestel, V., 2015	CT, Belgium, Cyprus, Estonia, Germany, Hungary, Italy, Spain, Sweden, long term	male Age$_{IG}$=6.22 ± 1.81<br>female Age$_{IG}$=6.26 ± 1.79<br>male Age$_{CG}$= 6.12 ± 1.73<br>female Age$_{CG}$=6.25 ± 1.70,<br>nIG= 3845<br>nCG=3568	Intervention<br>- Community level: involvement of community partners, long term media campaign and public relation strategies, lobbying for community environmental and policy interventions	no information provided	effectiveness of the IDEFICS intervention on objectively measured PA and SB	objective (accelerometer), sedentary time	0, not significant											1	2	7	0

		(51,9% male), not setting oriented	- School level: building partnerships, education of children (8 healthy weeks), environmental changed related to PA- the active playground, health related PE curricula<br>- Family level: education of parents (materials)																		
Verloigne, M., 2015	cluster RCT, Belgium, short term	Age=10.9±0.7, n_{IG}= 155 (45% male) n_{CG}=199 (38% male), School	Intervention<br>- Obtain objective data on sedentary time<br>- Teachers were encouraged to perform an activity break after every school hour Every intervention week covered one specific theme	no intervention	average time spent in sedentary bouts of 10 min or more among 10- to 12-year-old Belgian children	objective (accelerometer), (1) number of breaks during school hours, (2) number of sedentary bouts during school hours, (3) after school hours sedentary bouts >10 mins	⊕♀; results are only significant for girls: (1) β= -22.03 breaks (CI -34.37, 9.68) (2) β= 0.90 bouts in school (CI 0.14, 9.68) (3) β= -3.60 bouts >10 mins after school (CI -9.59, -0.61)											4	2	4	0
Weaver, R. G., 2018	CT, USA, long term	Age_{IG}=6.7-8.0years, n_{IG}= 54 (51,9% male) n_{CG}=97 (58,4% male), School	Intervention<br>- Increasing physical activity through schools	no intervention	increasing children's MVPA and decreasing sedentary time	objective (accelerometer),sedentary time	⊕♂; ♂$_{IG\ vs.\ CG}$=-11.8 % (CI 14.4 %, 1.9 %) ♀$_{IG\ vs.\ CG}$= +4.4 % (CI 4.7 %, 12.6 %)											2	2	6	0

Weaver, R. G., 2017	cluster RCT, USA, moderate term	Age_{IG}=7.9±2.0<br>Age_{CG}= 8.0±1.8,<br>n_{IG}= 904 (53,7% male)<br>n_{CG}=926 (56,4% male),<br>not Setting oriented	Intervention<br>- PA schedules; extend existing PA opportunities to three hours per day<br>- Encourage to expand PA opportunities by integrating short activity breaks and exchange inactive field trips for more activity<br>- Staff with professional development training covering the topic of enhancing the amount of activity in children (2 booster sessions)	Control Group received support for improving the types of food	increase the percentage of children meeting the 60 min/d MVPA guideline	objective (accelerometer), sedentary min/ day	⊕♀; group-by-time-interaction = -13.2 (CI -25.2, -1.2) group-by-time-interaction p value= 0.032											1	2	6	1

Note: ⊕⊕=positive intervention effect; favoured both ♂ and ♀; ⊕= positive intervention effect; favoured only one ♂ or ♀; 0=no intervention effect; = detailed; = basic; = no information provided; = poor
