# Supplementary material for: A Sex/Gender Perspective on Interventions to Reduce Sedentary Behaviour in Girls and Boys: Results of the genEffects Systematic Review
Source: Int J Environ Res Public Health. 2020 Jul 20;17(14):5231. doi: 10.3390/ijerph17145231 (PMC7400439; doi:10.3390/ijerph17145231)
Supplement: Supplementary file 1 [file ijerph-17-05231-s001.zip › Table S4_interaction SB studies.pdf]

Table S4. Summary of SB studies with interaction analysis for sex gender

Author, Year	Design, Country, Duration of the intervention	Age (M±SD), Sample Size (nIG (%male) nCG(%male)), Setting	intervention description	task of control group	aim of the study	SB measurement (measure used), outcome reported	Intervention outcome	Sex/gender checklist rating of items													
								1	2	3	4	5	6	7	8	9	10				
Annesi, J. J., 2015	RCT, USA, short term	Age=9.7 ±0.8, nCG=46, School	Intervention<br>- Standard Intervention: counsellors with 5 hours of training; instructor manual, participant workbook, 30-35min PA via non-competitive games<br>- Revised intervention: newly designed training of counsellors, strength training component, behavior and nutrition topics were reinforced, 30-35min sessions for PA; 35-40min sessions of MVPA, separate training manual for 5-8 year old and 9-12 year old children	no intervention	moderate-to-vigorous physical activity (MVPA)	objective (accelerometer), sedentary time	0, not significant											3	0	7	0
Duncan, S., 2011	cluster RCT, New Zealand, short term	9-11years, nIG=57(38.6 % male) nCG=40(32.5 % male), School	Intervention<br>- Each child received homework booklet organized into 5 PA topics<br>- Tasks were designed to encourage parental participation and family involvement<br>- 3 homework options for PA; children were required to complete at least one task per week for PA/week (e.g., walking from and to school)<br>- In-class exercises for three 1.5 hours session each week	no intervention	physical activity and dietary behaviours	subjective (diary), screen time	0, not significant											3	1	6	0

Engelen, L., 2013	RCT, Australia, moderate term	Age=5-7years n=221 (53.85% male), School	Intervention<br>- Materials were introduced on the school playgrounds to be used during all break times<br>- The adults participated in small groups to examine their own experiences of free play and the benefits and risks associated with active free play	no intervention	to increase children's physical activity during break time at school through active free play	objective (accelerometer), sedentary activity	0, not significant											3	1	6	0
Filho, V. C. B., 2016	cluster RCT, Brazil, moderate term	Age=11-18years, n_{CG}=637 (51,2% male), School	Intervention<br>- Training and activities in general curriculum<br>- PE teacher-specific training<br>- Opportunities in the school environment to engage in PA<br>- Health education in school community (e.g. posters)	no intervention	to assess the effect of a school-based intervention on PA-related variables among students	subjective (questionnaire), inactive students (	0, not significant											3	1	6	0
Harrison, M.,, 2006	CT, Ireland, moderate term	Age_{IG}=10.2±1.2 Age_{CG}=10.3±0.8, n_{IG}=182 (56% male) n_{CG}=130 (58% male), School	Intervention<br>- More opportunities to be physically active during school breaks, at noon or after-school hours<br>- Extra PA on after-school hours and at noon<br>- Extra sport materials (box)<br>- Personal PA advice<br>- Parents interactive group meeting on PA and relationship with health	no intervention	targeted sedentary behaviours and physical activity	subjective (recall), screen time	0, not significant											2	1	7	0
Kobel, S., 2014	cluster RCT, Germany, moderate term	Age_{IG}=7.06±0.63 Age_{CG}=7.06±0.63, n_{IG}=954 (59.8% male) n_{CG}=782 (52.6%	Intervention<br>- Materials offering action alternatives for recreational activities (without screen media)<br>- PA integrated in primary school curriculum	no intervention	increase of physical activity, a decrease in screenmedia time, reduction of the	subjective (questionnaire), screen media consumption	0, girls in the intervention group significantly used less screen media per day than their counterparts											3	1	6	0

		male), School			consumption of sugar-sweetened beverages		in the control group at baseline (OR= 0.58, p= 0.04, CI 0.35; 0.96)														
Laukkannen, A., 2015	cluster RCT, Finland, moderate term	Age_{IG}=6.07±1.12 Age_{CG}=6.20±1.13, n_{IG}=46 (45.7% male) n_{CG}=45 (46.7% male), Home	Intervention<br>- Provide instructions (outdoor PA, PA with peers…)<br>- Provide information on consequences (lecture, information about how PA enhances health)<br>- Prompting identification as a role model (information of concrete situations where parents act as physically active role model)<br>- Provide general encouragement and progressive goal setting	no intervention	increasing PA in children	objective (accelerometer), PA	0, not significant											3	1	6	0
Mendez-Gimenez, A., 2017	cluster RCT, Spain, short term	Age=10.64±1.13, n_{IG}=74 (48.65% male) n_{CG}=72 (52.78%), School	Intervention<br>- School with recreational period of 30 min in the morning every day of the week<br>- During the five days of one week, intervention group children were allowed to play freely during recess with the material they each had built	no information provided	to analyze whether an intervention increases the level of PA of students during recess	accelerometer (objective) ,sedentary activity	0, decrease between the pre-test and post-test in the experimental group in SB was similar in boys and girls (F=1,78)											2	1	6	1
Robinson, T.N., 2006	cluster RCT, USA, moderate term	Age_{IG}=8.9±0.6 Age_{CG}=8.9±0.7, n_{IG}=92 (55.4% male) n_{CG}=89 (42.8% male), School	Intervention<br>- After-school PA club<br>- Face-to-face motivational, individually tailored counseling session with registered nurse during the school day	no intervention	reducing children's time with individual screen media, effects of television viewing among parents	subjective (questionnaire), TV Viewing and video games weekday (hrs. /day), Saturday	⊕♂= Reductions in television were greater among boys than among girls (p= 0.05)											3	1	6	0

					and other household members																
Taylor, S. L., 2018	RCT, UK, short term	Age=9-10years, nIG=117 nCG=115, School	Intervention<br>- Activity cards with pictures demonstrating the activity and instructions on the back<br>- Instructor led high-intensity motor skills set to<br>- contemporary music, designed to improve<br>- health-related and skill-related fitness	no intervention	children's PA and sedentary time, promote PA across the school day	objective(accelerometer), sedentary time	0, not significant											0	2	7	1

Note: ⊕⊕=positive intervention effect; favoured both ♂ and ♀; ⊕= positive intervention effect; favoured only one ♂ or ♀; 0=no intervention effect; = detailed; = basic; = no information provided; = poor
