# Supplementary material for: A Sex/Gender Perspective on Interventions to Reduce Sedentary Behaviour in Girls and Boys: Results of the genEffects Systematic Review
Source: Int J Environ Res Public Health. 2020 Jul 20;17(14):5231. doi: 10.3390/ijerph17145231 (PMC7400439; doi:10.3390/ijerph17145231)
Supplement: Supplementary file 1 [file ijerph-17-05231-s001.zip › Table S5_sex_gender tested SB studies.pdf]

Table S5. Summary of sex/gender tested SB studies

**Author, Year**	**Design, Country, Duration of the intervention**	**Age (M±SD), Sample Size (nIG (%male) nCG(%male)), Setting**	**intervention description**	**task of control group**	**aim of the study**	**SB measurement (measure used, outcome reported)**	**Sex/gender checklist rating of items**													
							1	2	3	4	5	6	7	8	9	10				
Adab, P., 2018	cluster RCT, UK, long term	Age$_{IG}$=6.3±0.3<br>Age$_{CG}$=6.3±0.3,<br>n$_{IG}$=662 (49,2% male)<br>n$_{CG}$=735 (52,7% male),<br>School	Intervention<br>- Helping teachers to provide opportunities for additional MVPA during school day<br>- Participation in "Villa Vitality" programme, delivered through sport institution<br>- Information sheets to families signposting PA opportunities	no intervention	preventing obesity	objective (accelerometer), sedentary time											3	1	6	0
Andrade, S., 2014	cluster RCT, Ecuador, long term	Age$_{IG}$=12.8±0.2<br>Age$_{CG}$=12.9±0.3,<br>n$_{IG}$=700<br>n$_{CG}$=740(33,6% male),<br>School	Intervention<br>- Reading books with information about PA<br>- Parental workshops to increase activity behavior<br>- Social events, walking trial and posters with information about healthy behaviours	no intervention	physical fitness, screen time, PA, BMI	objective (accelerometer), sedentary time, subjective (questionnaire) Screen time in week day and weekends											3	1	6	0
Breslin, G., 2012	cluster RCT, UK, short term	Age$_{IG}$=9.12±0.37<br>Age$_{CG}$=9.09±0.35,<br>n$_{IG}$=209<br>n$_{CG}$=207,<br>School	Intervention<br>- PA and healthy eating programme, based on social cognitive theory	no intervention	physical activity, decrease sedentary behaviour, screen time, nutrition in 8-9-year old school children from socially and economically disadvantages areas of Northern Ireland	subjective (questionnaire),watching TV,DVD, playing video games											2	1	7	0

Cradock, A. L., 2016	cluster RCT, USA, moderate term	Age_{IG}=7.9±1.8<br>Age_{CG}=7.6±1.6,<br>n_{IG}=182 (46,7% male)<br>n_{CG}=220 (50,5% male),<br>School	Intervention<br>- to include 30 minutes of moderate, fun PA for every child every day<br>- to offer 20 min of VPA 3 times per week<br>- strategies to incorporate PA breaks and small-space activities into the program	wait-listcontrol group	physical activity levels in existing after-school programs.	objective (accelerometer),sedentary behavior											3	1	7	0
De Coen, V., 2012	cluster RCT, Belgium, long term	Age_{IG}=4.86±1.25<br>Age_{CG}=5.04±1.29,<br>n_{IG}=1280(52,9% male)<br>(45,3% male),<br>School	Intervention<br>- screen behavior (hours of daily screen-time)<br>- nutritional and PA<br>- development of active playground, implementation of health-related PE<br>- Environmental and policy changes to increase availability of water at school<br>- implement 5 Healthy weeks / intervention year	no information provided	BMI, eating and PA in Flanders	subjective (questionnaire),Screen-time (h/day)											1	1	7	1
Fairclough, S. J., 2013	cluster RCT, UK, moderate term	Age=10.6±0.3years,<br>n_{IG}=166<br>n_{CG}=152,<br>School	Intervention<br>- Teachers received 4 hours of training in the delivery of curriculum resource<br>- Worksheets, weekly lesson plans, homework tasks, lesson resources, CD-ROM<br>- Key component: "Move more and sit less"	no intervention	measures of body size, PA and food intake	objective (accelerometer),Sedentary time (min/day)											1	1	7	1
Lanckriet, S. , 2017	cluster RCT, France, moderate term	Age_{IG}=9.97±0.86<br>Age_{CG}=9.94±0.83,<br>n_{IG}=73 (45,2% male)<br>n_{CG}=107 (51,4% male),<br>School	Intervention<br>- Action groups with two recreation sessions of 45min per week using kit (consists of a box contains the equipment needed to play)	no intervention	children's level of PA	objective (accelerometer), playing video games											3	0	7	0

Ni Mhurchu, C., 2008	cluster RCT, New Zealand, short term	Age=12±1.5years, n_{IG}=10<br>n_{CG}=10 (60%male),<br>Home	Intervention<br>- Active video games; substitute usual non active video game play with active video games	no intervention	effect of active video games on children's PA	objective (accelerometer), PA											2	0	7	1
Nyberg, G., 2016	cluster RCT, Sweden, moderate term	M=6.3±0.3years, n_{IG}=185 (48,1% male)<br>n_{CG}=196 (50,8% male),<br>School	Intervention<br>- Health information brochures for parents<br>- Motivational interviewing<br>- Classroom activities (target children's knowledge, attitudes and preferences and parent's role modelling)	no intervention	dietary habits, physical activity and body weight of six-year-old children in families with low socio-economic status in the school context	objective (accelerometer), sedentary time during weekday and weekend											2	1	7	0
O'Dwyer, M. V., 2012	cluster RCT, UK, short term	M=3.8±0.6years, n_{IG}=34<br>n_{CG}=43,<br>Health care	Intervention<br>- 70min sessions (10min registration and checking home activity completion, 60min delivery time)<br>- Children: active play, parents: educational workshop (first 20 of 60min)<br>- Active play (parents and children together; 40min)<br>- Log book	no interventi on	SB (weekend and weekday), PA	subjective(questionnaire), MVPA, TV watching in weekdays-weekend											2	2	5	1
Smpokus, E. A., 201	cluster RCT, Greece, moderate term	M=7.6±0.9years, n_{IG}=359(52,1% male)<br>n_{CG}=439(50,3% male),<br>School	Intervention<br>- Educational program was not described in detail	no information provided	physical activity and fitness indices	objective (accelerometer), sedentary time											1	2	6	1

Toftager, M., 2014	cluster RCT, Denmark, long term	Age_{IG}=12.5±0.63<br>Age_{CG}=12.5±0.61,<br>n_{IG}=376 (48,7% male)<br>n_{CG}=421 (52,7% male),<br>School	Intervention<br>- Upgrade existing outdoor areas at the school for PA<br>- Develop and build playgrounds<br>- improve safety for active transport<br>- Establish an after school fitness program<br>- Formulate and implement school PA policy<br>- Educate teachers as ''kick-starters''<br>- Establish school play patrol: older students were trained to initiate play and games for minors during school recess<br>- Mandatory outdoor recess<br>- School traffic patrol: older students helped minors cross the streets near the school<br>- Educate and train students in safe cycling,<br>- School project/theme week once a year focusing on learning about and doing PA during school lessons	no informati on provided	reducing the decline in PA among students(11-15 years)	objective (accelerometer), sedentary time											3	2	5	0
Vik, F. N., 2015	cluster RCT, Five European countries: Belgium, Germany, Greece, Hungary, Norway , short term	Age=11.2 years,<br>n_{IG}=1569<br>n_{CG}=1578,<br>School	Intervention<br>- aiming at reducing and breaking up sitting time at home (with special emphasis on screen time), and breaking up sitting time in school among 10 – 12 year olds in Europe, evaluate its short term effects	no interventi on	reducing screen time, increasing breaking up sitting time,changing specific personal determinants	subjective(questionnaire), objective(acceleromet er),breaking up sitting time											3	1	6	0

Note: = detailed; = basic; = no information provided; = poor
