# Supplementary material for: A Sex/Gender Perspective on Interventions to Reduce Sedentary Behaviour in Girls and Boys: Results of the genEffects Systematic Review
Source: Int J Environ Res Public Health. 2020 Jul 20;17(14):5231. doi: 10.3390/ijerph17145231 (PMC7400439; doi:10.3390/ijerph17145231)
Supplement: Supplementary file 1 [file ijerph-17-05231-s001.zip › Table S6_single sex_gender SB studies.pdf]

Table S6. Summary of single sex/gender SB studies

Author, Year	sex/gender of participants	Design, Country, Duration of the intervention	Age (M±SD), Sample Size (nIG (%male) nCG(%male)), Setting	intervention description	task of control group	aim of the study	SB measurement (measure used), outcome reported	Intervention effect	Sex/gender checklist rating of items														
									1	2	3	4	5	6	7	8	9	10					
Bakhoya, M., 2016	girls	cluster RCT, USA, moderate term	Age=11.97±0.70, n_{CG}=64, School	Intervention<br>- Booster intervention with 13 motivational, individual tailored postcards, enclosed in bright pink envelopes mailed to each girl's home during postintervention<br>- Each postcards included: fun activity about PA for girls to complete and a motivational, individually tailored message to encourage PA<br>- Messages were tailored based on each girl's personal responses to iPad survey	no intervention	moderate-to-vigorous PA	objective (accelerometer), time effect moderate-to-vigorous physical activity	0; p=0.898											0	0	6	0	4
Carlin, A., 2018	girls	cluster RCT, UK, short-moderate term	Age=12.4±0.6, n_{IG}=101 n_{CG}=98, School	Intervention<br>- Participants were provided with the opportunity to attend a number or structured 10-15min walks spread across the school week before the first bell	no intervention	school-time PA and sedentary behaviour	objective (accelerometer), sedentary (min/school day)	⊕ ♀; M=−8.82 (CI −13.99; -3.64)											2	0	4	0	4

				- Walks were at moderate PA intensity Schools were instructed to provide at least 2 walking sessions for participants to attend each day																			
Dewar, D. L., 2014	girls	cluster RCT, Australia, moderate term; 12 months	Age=13.2± 0.5, n_{IG}=178 n_{CG}=179, School	Intervention<br>- School sport sessions, interactive seminars, nutrition workshops, lunch-time PA, PA and nutrition handbooks, parent newsletters, pedometers for self-monitoring, text messaging for social support	no intervention	improving physical activity, dietary and sedentary behaviors	subjective (self-report), computer use, sedentary behavior	⊕♀; M= −26.0 (CI −46.9; −5.1, p = 0.02)											1	1	4	0	4
Fairclough, S. J., 2006	girls	CT, UK, short term; 5 weeks	Age=11-12years, nIG=30-32 nCG=30-32, School	Intervention<br>- Teachers received 4 hours of training in the delivery of curriculum resource<br>- Worksheets, weekly lesson plans, homework tasks, lesson resources, CD-ROM<br>- Key component: “Move more and sit less”	no intervention	to increase cardio respiratory health-enhancing PA levels during girls' gymnasitc lessons	objective (observartion),Lying down, sitting, standing	0; p=0.13											1	0	5	0	4

Jago, R.,, 2006	boys	RCT, UK, short term	Age_{IG}=13±0.1<br>Age_{CG}=13±0.1,<br>n_{IG}=240<br>n_{CG}=233,<br>Community	Intervention<br>- Program of peer-led, teacher-facilitated learning activities known as FLASH modules (Fun Learning Activities for Student Health)<br>- Modules contained sessions designed to foster self-awareness, knowledge, decision-making skills, peer involvement for health behaviour change<br>- Social marketing campaign (e.g., encouraging PA)<br>- More active PE curriculum	control group received a "mirror image" fruit and vegetable intervention	effect of a Boy-Scout-based physical activity intervention	objective (accelerometer), sedentary minutes/ day	⊕ ♂;M= -12											1	1	4	0	4
Jago, R.,, 2015	girls	cluster RCT, UK, moderate term	Age=11-12years,<br>n_{IG}=273<br>n_{CG}=276,<br>School	Intervention<br>- Teaching assistants with training programme that focusses on delivering PA programme in after-school environment<br>- Action 3:30 club with detailed session plans<br>- Equipment for each intervention school to provide additional resources for Action 3:30 club	Girls in the control schools provided data only.	increase the time spent in weekday MVPA among Year 7 girls	objective (accelerometer), weekday sedentary mins	0; M=-6.79 (CI -23.60; 10.03)											3	1	2	0	4

Lubans, D. R., 2012	girls	RCT, Australia, moderate term	Age_{IG}=13.15±0.44 Age_{CG}=13.20±0.45 , n_{IG}=178 n_{CG}=1797, School	Intervention<br>- Influences on physical activity and nutrition behavior change<br>- Promoting lifetime physical activities, reducing sedentary behaviors, and encouraging low-cost healthy eating<br>- All intervention schools were provided with a standard equipment pack (value=US $1300), which consisted of a range of equipment	wait-list-control group	preventing obesity	objective (accelerometer), daily screen time min/d	⊕ ♀; M= -30.67 (CI -62.43; -1.06)											2	1	3	0	4
Lubans, D. R., 2016	boys	cluster RCT, Australia, moderate term	Age_{IG}=12.7±0.5 Age_{CG}=12.7±0.5, n_{IG}=181 n_{CG}=180, School	Intervention<br>- Explicitly targeting resistance training movement skill competency<br>- Teachers were trained to deliver the enhanced sport sessions using the SAAFE (Supportive, Active, Autonomous, fair and Enjoyable) teaching principles, designed to enhance students' autonomous motivation for physical activity	wait-list-control group	Improve the autonomous motivation for school sport and leisure-time physical activity	subjecive (questionnaire), screen-time min/day	⊕ ♂; M=−32.2 (CI −53.6; −10.8)											2	0	3	1	4

Morgan, P.J., 2018	girls	RCT, Australia, short term	Age=7.7years, n_{IG}=74 n_{CG}=79, Community	Intervention<br>- Combined education session: Father-only education session, daughter-only education session Combined practical session<br>- Sport skills programm; sports equipment pack; DADEE app	wait-list-control group	improve the physical activity levels of fathers and their daughters	objective (pedometer), screen-time for daughters	⊕ ♀; Mean difference between groups= 0.5–0.8											4	1	1	0	4
Okely, A. D., 2017	girls	cluster RCT, Australia, long term	Age=13.6±0.02, n_{IG}=566 n_{CG}=633, School	Intervention<br>- Formula curriculum: enhance school sports by trying to engage girls for at least 50% of class rime<br>- School environment: e.g. after-school activity programs, modifying school policies related to use of equipment<br>- Links with community: promotion out of school activities (e.g. use local facilities – fitness centres)<br>- Meetings with a member of the research team<br>- Funding and professional development activities (2-day	wait-list-control group	slow the decline in physical activity (MVPA) among adolescent girls	objective (accelerometer), sedentary activity, mins/day	0; M=-2.81 (CI -21.74; 16.19)											2	1	3	0	4

				training program and 2-day research symposium)																			
Rosenkranz, R. R., 2010	girls	cluster RCT, USA, moderate term	Age=10.5, nIG=33 nCG=39, Commuity	Intervention<br>- Educational curriculum (e.g. target behaviors, worksheet for goal setting and self-monitoring, PA recreation session etc.)<br>- Troop leader training<br>- Support by intervention staff (e-mail and phone support)	no intervention	prevention of obesity	objective (accelerometer), sedentary behavior	⊕ ♀; $x^2 = 6.3$ (p= 0.011)											1	1	3	1	4
Sebire, S.J., 2018	girls	cluster RCT, UK, short term	Age=12-13years, n_{IG}=269 n_{CG}=158, School	Intervention<br>- Trainers with three-day train-the-trainers programme approximately one week before delivery<br>- Peer-supporter training was provided for each school to the top of about 18 girls nominated to be a peer-supporter and delivered by two trainers; peer-supporter returned to school and informally diffused messages about PA to friends	no intervention	week-day physical activity	objective (accelerometer), sedentary time (min/weekday)	⊕ ♀; M=-23 (CI -43.73; -2.79)											1	1	3	1	4

Smith, J. J., 2014	boys	cluster RCT, Australia, moderate term	Age_{IG}=12.7±0.5 Age_{CG}=12.7±0.5, n_{IG}=181(100% male) n_{CG}=180(100% male), School	Intervention<br>- Teachers professional development (workshops)<br>- Parental newsletters (information about potential consequences of excessive screen use among youth, strategies for reducing screen-based recreation in family home, tips for avoiding conflict when implementing rules)<br>- Researchers-led seminars for students (seminars that provide information)<br>- Enhanced school-sport sessions<br>- Lunchtime PA mentoring sessions<br>- Smartphone app and website (PA mentoring)<br>- Pedometers	wait-list-control group	school-based obesity prevention intervention for adolescent boys	subjective (questionnaire), screen time min/day	⊕ ♂; M= -30 (SE= 10.08; p= 0.03)											3	1	2	0	4
Young, D. R., 2006	girls	RCT, USA, moderate term	Age=13.8±0.5, nIG=111 nCG=99, School	Intervention<br>- Make an informed decision about<br>- the personal benefits of a physically active lifestyle, develop problem-solving skills, and obtain	Parents of participants in the standard PE class also received	increasing physical activity and fitness	subjective (questionnaire), ≥ 3 hour of watching television school day, 3 h of playing video games or using	⊕ ♀; proportion of participants spent ≥3 hrs TV on school days d_{IG}=5,3 % (p=.03)											2	1	4	0	3

				support from others<br>- Skills were taught using class lectures and discussions, small-group discussions, and home<br>- work activities<br>- Students were encouraged to keep weekly exercise logs Teaching units that were active in nature, breaking skills training into small-group activities, and playing games in small groups<br>- Family workshop, monthly newsletters, and adult-child homework assignments.	monthly newsletters. Topics were of general health interests and included an article about PE class content that month.		the internet school day																

⊕= positive intervention effect; favoured only one ♂ or ♀; 0=no intervention effect; = detailed; = basic; = no information provided; = poor, = not relevant
